# Exotic cuticular specializations in a Cambrian scalidophoran

**DOI:** 10.1098/rspb.2024.2806

**Published:** 2025-02-05

**Authors:** Giovanni Mussini, Nicholas J. Butterfield

**Affiliations:** ^1^ Department of Earth Sciences, University of Cambridge, Downing Street, Cambridge CB2 3EQ, UK

**Keywords:** Cambrian explosion, priapulid, scalidophora, morphological disparity, ecdysozoa

## Abstract

Scalidophora, the ecdysozoan group including priapulids, kinorhynchs and loriciferans, comprises some of the most abundant and ecologically important Cambrian animals. However, reconstructions of the morphology and lifestyles of fossil scalidophorans are often hampered by poor preservation of their submillimetre-scale cuticular specializations. Based on exceptionally preserved small carbonaceous fossils (SCFs), we describe a new scalidophoran-grade animal, *Scalidodendron crypticum* gen. et sp. nov., from the Early to Middle Cambrian Hess River Formation of northern Canada. The Hess River SCFs comprise pharyngeal teeth, coniform sclerites and hook-like sclerites, all closely comparable to known scalidophoran counterparts. The coniform and hook-like sclerites recurrently associate with arborescent cuticular projections that show multiple orders of branching, morphologically unlike those of any known living or fossil scalidophoran. The fine splintering and inferred post-pharyngeal position of these structures argue against locomotory, feeding and defensive roles with direct analogues in extant counterparts. As such, the arborescent structures of *Scalidodendron* denote a previously cryptic range of morphological variation in Cambrian scalidophorans, paralleling that of coeval panarthropods but expressed at a fundamentally different level of anatomical organization.

## 1. Introduction

Scalidophora is a group of ecdysozoan animals comprising three phyla: the vermiform proboscis-bearing priapulids, the segmented, spinose kinorhynchs and the meiofaunal corselet-bearing loriciferans [1–4]. These phyla may form a monophyletic group, representing the sister-taxon to either nematoids or a clade encompassing both nematoids and panarthropods [5–7]. Alternatively, priapulids and kinorhynchs may record successive outgroups to all other ecdysozoans, with loriciferans as the sister group of nematoids [6,7]. Regardless of their phylogenetic placement, all scalidophorans share a set of distinguishing features including an eversible introvert, an annulated trunk, a network of circular, longitudinal and retractor muscles, and cuticular sclerites expressing a range of spinose, flap-like and tubular morphologies, including head ‘scalids’ and posterior spines and/or plates on the trunk region [1–3,8].

With little over 400 described species, scalidophorans constitute minor components of modern animal biodiversity. By contrast, they appear to have been the most diverse and abundant group of Cambrian endobenthic worms [9,10]. Priapulid-grade ecdysozoans are prevalent in macrofossil Lagerstätten including those of Chengjiang [9], Sirius Passet [11] and the Burgess Shale [12]*,* where they may have been ecologically important bioturbators and nutrient cyclers [10,13]. Loriciferans and kinorhynchs are also known from multiple Cambrian deposits as carbonaceous and phosphatized fossils. These include the possible stem-kinorhynch *Eokinorhynchus* [4] from the lowermost Cambrian (approx. 535  Ma) Kuanchuanpu Formation, *Sirilorica* from the early Cambrian Sirius Passet biota [14] and the unambiguous loriciferan *Eolorica* from the late Cambrian Deadwood Formation [15].

Despite this relatively diverse fossil record, the submillimetric sclerites and cuticular specializations of Cambrian scalidophorans are often obscured by taphonomic compression [3,16], severely hampering the reconstruction of their morphological and functional variability [17]. This taphonomic barrier can be partly overcome by small carbonaceous fossils (SCFs) [18]: organic micro- to mesofossil that permit micrometre-scale resolution of cuticular structures, free from the host matrix and adpressed body parts [16]. Isolated scalidophoran SCFs have revealed cuspidate (spear-shaped) pharyngeal teeth, introvert hooks and subconical sclerites, all of which find at least approximate morphological and functional counterparts among extant taxa [15,17,19,20]. These structures represent only a minor component of the ecdysozoan repertoire of cuticular specializations, which encompasses both spine- and tooth-like sclerites and nonsclerotized hairs and papillae [21]. The relatively modest level of morphofunctional variation in Cambrian scalidophoran cuticles stands at odds with that of coeval panarthropods bearing arrays of spinose, plated and setulose limbs and tagmata falling outside the range of extant counterparts [22–24], most notably the cuticular armours and suspension feeding apparati of stem-onychophorans [22].

Here, we describe the first record of similarly ‘exotic’ cuticular structures in Cambrian scalidophoran-grade animals, from a SCF assemblage recovered from offshore, slope facing mudstones of the Hess River Formation of northwestern Canada (Supplementary Materials, Geological Context; electronic supplementary material, figure S1). Their lack of close counterparts among known priapulids, loriciferans or kinorhynchs suggests a correspondingly unconventional function, and points to a novel category of cuticular specializations that expands the known morphological variability of Cambrian ecdysozoans.

## 2. Systematic palaeontology

Unranked clade Ecdysozoa Aguinaldo, 1997

Unranked clade Scalidophora Lemburg, 1995

*Scalidodendron crypticum* gen. et sp. nov.

### (a) Holotype

**Figure 1.**
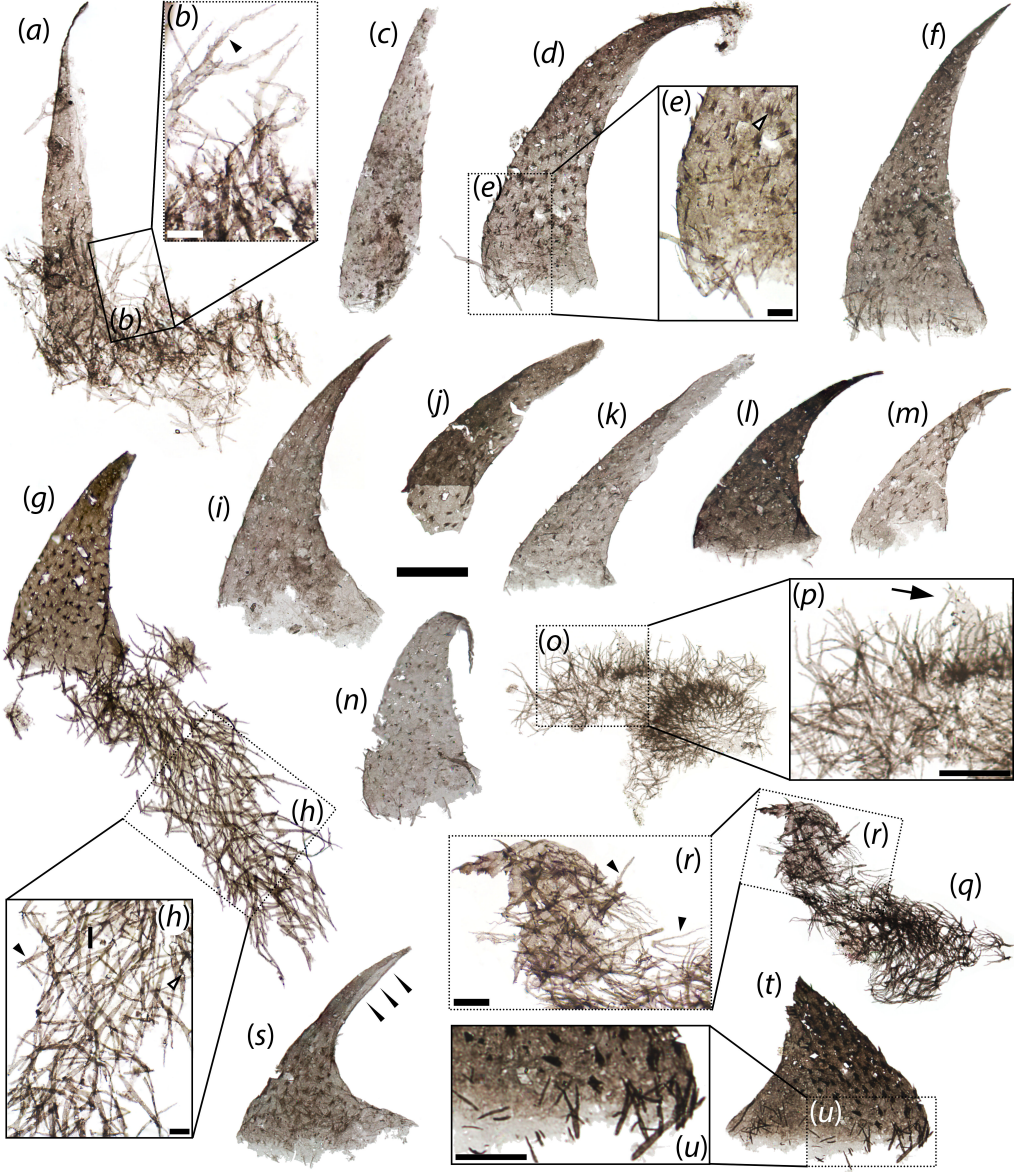
*Scalidodendron crypticum* gen. et sp. nov., hook-like sclerites. (*a*) Straight-sided hook with Type 2 arborescent structures basally. (*b*) Detail of arborescent structures in a, showing serrated margins (black arrowhead). (*c*) Incomplete spinulose hook. (*d*) ‘j-shaped’ hook. (*e*) Detail of d showing basal arborescent structures and comb-like surface projections (white arrowhead). (*f*) Elongate recurved hook with basal arborescent structures. (*g*) Stout recurved hook with extensive basal cluster of arborescent structures. (*h*) Detail of boxed area in g, showing possible lateral rods (black arrowhead) and serrated margin (white arrowhead). (*i*) Recurved spinulose hook. (*j*) Incomplete hook showing hollow internal structure basally. (*k*) Incomplete hook showing hollow internal structure distally. (*l-n*) Short hook-like sclerites showing sparse arborescent structures basally. (*o*) Dense cluster of Type 2 arborescent structures with short spinose sclerite. (*p*) Detail of boxed area in o, showing short, erect spinose sclerite surrounded by Type 2 arborescent structures. (*q*) Cluster of Type 2 arborescent structures surrounding a markedly recurved sclerite with long surface spines. (*r*) Detail of boxed area in q showing serrated margins (black arrowheads) and spinose sclerite. (*s*) Apically split sclerite (see black arrowheads) showing internally hollow construction. (*t*) Incomplete sclerite with spinose to arborescent projections basally. (*u*) Detail of spinose to arborescent cuticular protrusions in t. Slide numbers and England Finder coordinates provided in electronic supplementary material, data S1. Scale: 50 μm except in b, e, h, r (10 μm) and p, u (25 μm).

HR−3-h49 (figure 1*g*), a spinulose hook-like sclerite associated with arborescent structures.

**Figure 2.**
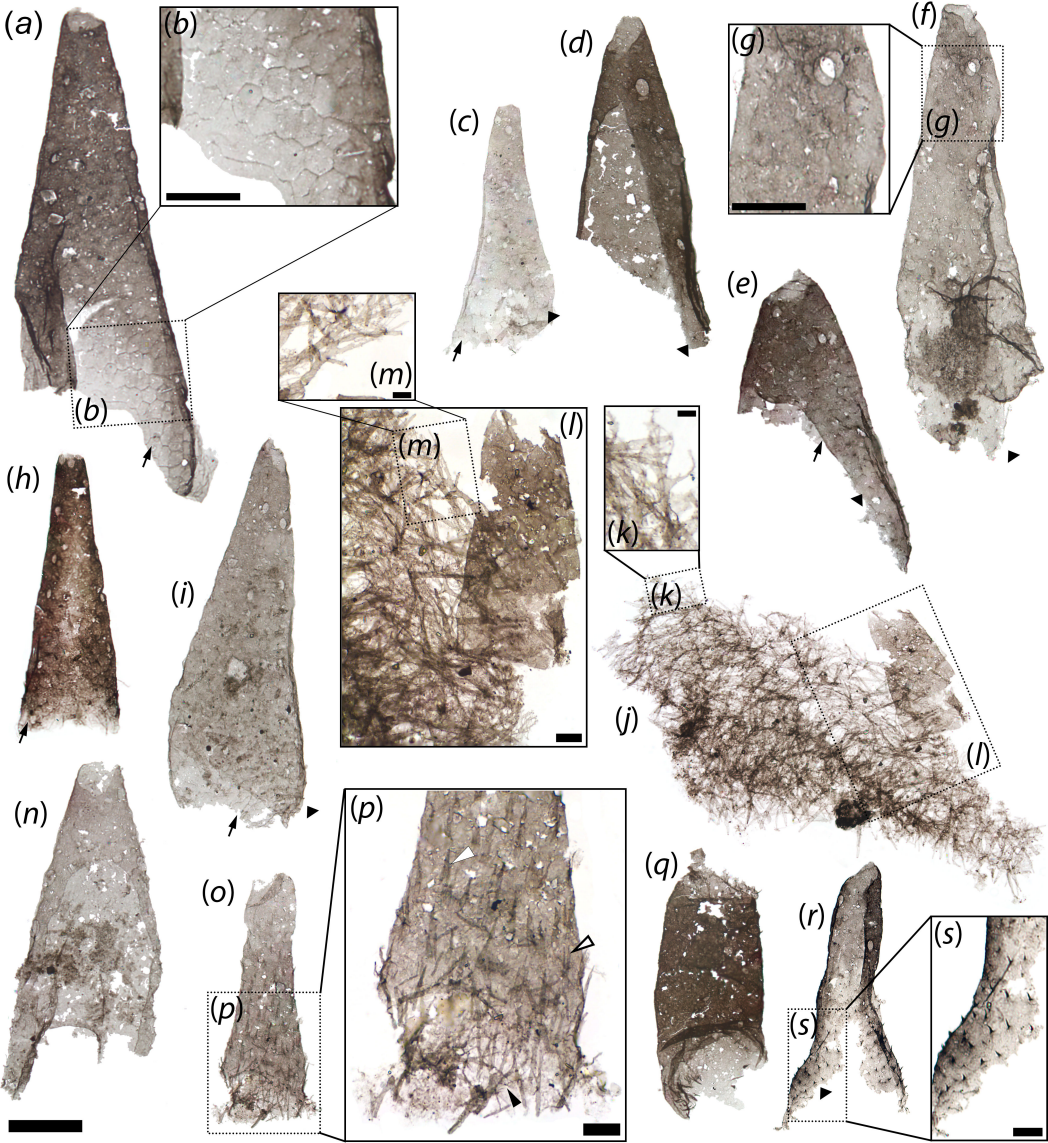
*Scalidodendron crypticum* gen. et sp. nov., coniform sclerites. (*a*) Large coniform sclerite with surface perforations. (*b*) Detail of boxed area in a, showing hexagonally patterned basal cuticle. (*c*) Thin-walled coniform sclerite showing apical perforation, polygonally patterned basal cuticle (black arrow) and spinules (black arrowhead). (*d-e*) Hollow coniform sclerite showing apical perforations, polygonally patterned basal cuticle (black arrow) and spinules (black arrowhead). (*f*) Hollow coniform sclerite showing basal and apical openings, apical ovoid perforations, corrugated cuticle and external spinules (black arrowhead). (*g*) Detail of boxed area in f showing apical perforations. (*h*) Straight coniform sclerite showing basal filiform projections and lateral rows of perforations; possible polygonally patterned cuticle is indicated by black arrow. (*i*) Thin-walled coniform sclerite with lateral perforations and possible polygonally patterned cuticle, indicated by black arrow. Short spinules are denoted by black arrowhead. (*j*) Mat-like aggregate of arborescent structures, with associated coniform sclerite laterally. (*k*) Detail of arborescent structure in j. (*l*) Detail of coniform sclerite and associated arborescent structures in j. (*m*) Detail of arborescent structures in l. (*n*) Hollow coniform sclerite showing basal and apical openings exposing internal surface. (*o*) Coniform sclerite with dense spinulose to bifid surface ornament. (*p*) Detail of spinulose (solid white arrowhead) to bifid (arrowhead with black margin) ornamentation in o, grading into arborescent projections (black arrowhead) basally. (*q*) Tubular sclerite with basal spinules. (*r*) Sclerite with lateral perforations and flaring base with spinulose ornament. (*s*) Detail of basal spinules in r. Slide numbers and England Finder coordinates provided in electronic supplementary material, data S1. Scale: 50 μm except for b, g (25 μm), l, p, s (10 μm).

### (b) Designated paratypes

**Figure 3.**
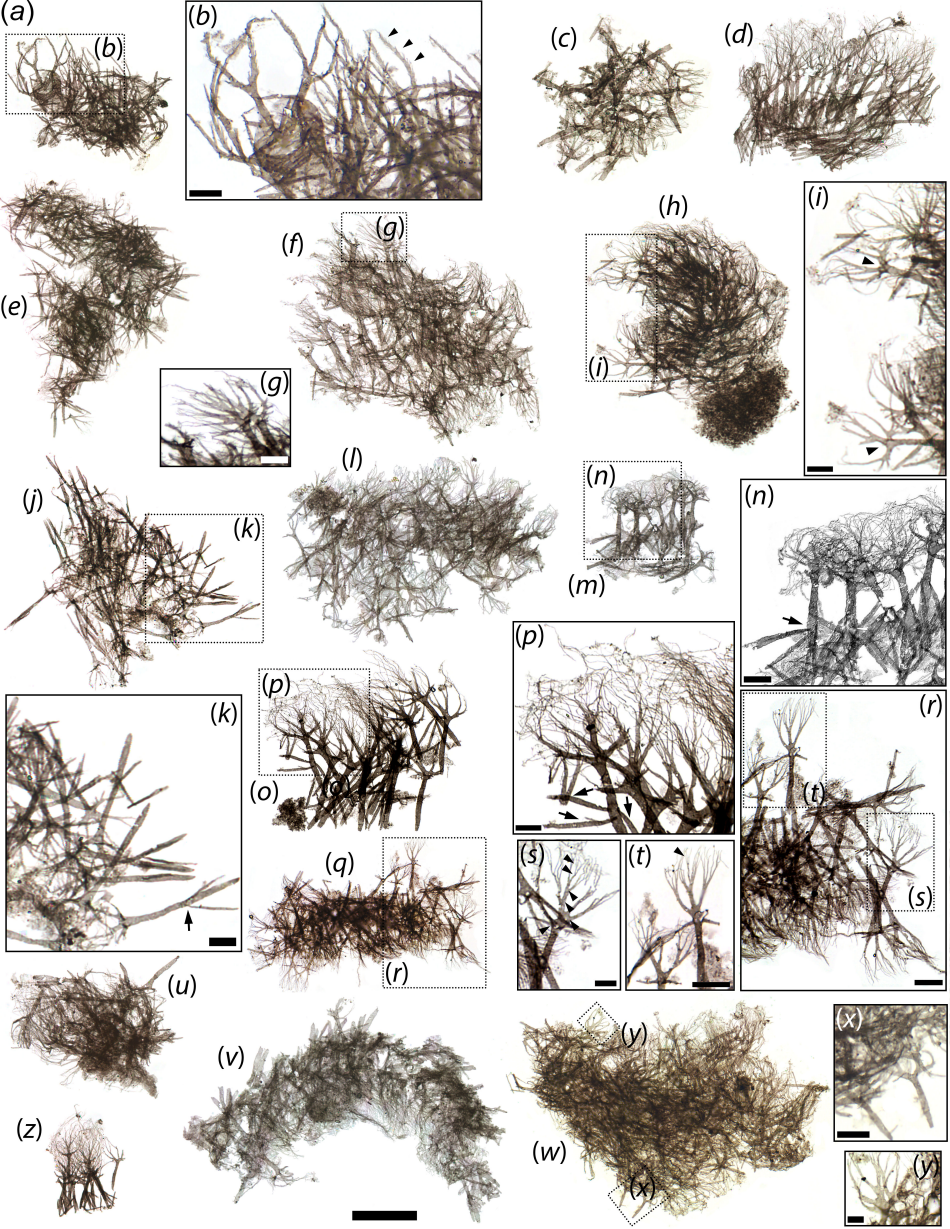
*Scalidodendron crypticum* gen. et sp. nov., arborescent structures. (*a*) Type 2 structures. (*b*) Detail of boxed area in a showing serrated margins (black arrowheads). (*c-f, h, j, l, h, n, o, q, u-w, z*) Type 1 structures. (*g*) Detail of boxed area in g showing palmate branching crown. (*i*) Detail of boxed area in h showing palmate branching crowns and splits between branches (black arrowheads). (*k*) Detail of boxed area in j showing laterally projecting rods (black arrow). (*n*) Detail of boxed area in m showing finely splintered crown and laterally projecting rods (black arrows). (*p*) Detail of boxed area in o showing palmate branched with long distal tendrils and lateral rods (black arrows). (*r*) Detail of boxed area in q. (*s*) Detail of boxed area in r, showing palmate branches with up to five subsequent orders of bifurcations (black arrowheads). (*t*) Detail of boxed area in r showing palmate branches and tripartite distal splintering. (*x-y*) Details of arborescent structures in w. Slide numbers and England Finder coordinates provided in electronic supplementary material, data S1. Scale: 50 μm except for *b, g, k, I, n, p, r, t, x* (10 μm), *s, y* (5 μm).

Forty one specimens, including 14 coniform sclerites (of which 3 are associated with arborescent structures), 12 hook-like sclerites (of which 8 associated with arborescent structures) and 15 clusters of arborescent structures without associated sclerites (electronic supplementary material, data S1). Figures 1a–f,i–u–2 and 2–3 (except 1*g*); slide numbers and England Finder coordinates provided in electronic supplementary material, data S1.

### (c) Referred material

Twenty two specimens: 3 priapulid-type tail hooks, 5 introvert hooks, 2 coronal spines, 1 isolated cluster of possible locomotory scalids, 2 possible disarticulated locomotory scalids and 9 pharyngeal teeth (electronic supplementary material, data S1) electronic supplementary material figures S2–S3; slide numbers and England Finder coordinates provided in electronic supplementary material, data S1.

### (d) Locality and horizon

Hess River Formation (64°36.324’N, 130°03.573’W), section height unknown (see §3).

### (e) Etymology

The genus name combines the root of the word ‘scalid’ (from the Greek *‘Scalidon’*, σκαλιδον, ‘hoe’), the etymological root of scalidophorans, with the Greek word ‘*Dendron*’ (δένδρον) for ‘tree’. This name refers to the taxon’s diagnostic arborescent structures.

The species epithet *crypticum* (Latin for concealed) refers to the problematic nature and possible camouflaging function of the arborescent structures.

### (f) Diagnosis for genus and species

Scalidophoran with coniform and hook-like sclerites bearing spinulose to comb-like projections on their outer surface. The hook-like and coniform sclerites are frequently found physically associated with arborescent cuticular structures. Each arborescent structure comprises a shaft with multiple apical branches, each splitting into up to five orders of finer distal projections. The tips of the distal projections consist of distally tapering straps with serrated lateral edges, or fine tendrils.

### (g) Description

*Scalidodendron* SCFs comprise two sclerite types: hook-like and coniform. These elements are united by their thin-walled cuticular construction, oval to subcircular cross-sections, spinulose ornament on their outer surface and exclusive association with arborescent structures (electronic supplementary material, data S1).

#### (i) Hook-like sclerites

*Scalidodendron*’s hook-like sclerites (*n* = 13) comprise a continuum of cuspidate elements approximately 100–250 μm long and approximately 70–100 μm wide at the base. The hook-like sclerites flare into a semi-elliptical base and taper to a fine point distally. Their apex is deflected from the perpendicular to the base at an angle ranging from less than 10° to nearly 90° in different specimens (figure 1*a*, *c*, *f*–g,i–n, cf. *d*, *l*, *r–t*,); accordingly, the hook-like sclerites range from nearly straight (figure 1*a*) to markedly falciform or bent (figure 1*l*,*r*). Whereas most display approximately constant curvature ratios along their entire length (figure 1*f*,*g*,*i*,*l*), one specimen bends sharply in its distalmost portion, yielding a somewhat J-shaped profile (figure 1*d*). The hook-like sclerites are hollow, as shown by their basal apertures and the visibility of superimposed, semi-transparent walls from opposite sides of the sclerite (figure 1*j*,*l*–m,s). The cuticular walls of the hook-like sclerites are thinnest basally and become more robust and optically dense apically. In one specimen, the cuticle along the concave margin is split, resulting in an apparent ‘cleft’ exposing the internal cavity and extending down from the apex for nearly half the length of the hook-like sclerites. This split, not observed in other specimens, likely represents a taphonomic artefact (figure 1*s*).

Approximately 30 to 120 cuticular spinules (figure 1*c–f,g,i–n,p,r–t*) or fine comb-like projections (figure 1*e*) are visible on the external surface of each hook. Although both types of surface ornaments may be visible on the same specimen, the comb-like projections tend to be confined to the basal portion of the hook-like sclerites (figure 1*e*, cf. k, m). Both types of projections tend to be regularly spaced and patterned into uneven proximo-distal rows along the length of the hook. The spinules are triangular, distally directed, and up to approximately 12 μm long and approximately 5 μm wide. Compared to the rest of the sclerite they consist of much darker and opaque material, suggesting greater sclerotization. The comb-like projections are up to approximately 8 wide and approximately 10 μm long. Each consists of a U-shaped cuticular flange bearing 4−5 symmetrically arranged bristles on its distal margin. The bristles point distally and comprise about half of the total length of each comb-like projection (figure 1*e*).

#### (ii) Coniform sclerites

The coniform sclerites of *Scalidodendron* (*n* = 14) are approximately 50–100 μm wide at the base and up to approximately 280 μm long. They resemble the co-occurring hook-like sclerites (figure 1) in their delicate outer wall and elliptical to subcircular cross-sections (figure 2). However, they are never falciform. Instead, they consist of hollow sclerites ranging from subcylindrical (figure 2*o–q*) to more markedly conical, with a nearly triangular transverse section (figure 2*a*,*d*,*h*,*n*). The coniform sclerites also show a rounded apical opening (figure 2*a,d–f,h–i,n*) instead of the cuspidate termination characterizing hook-like counterparts (figure 1).

The outer walls of the coniform sclerites are generally smooth, with occasional folds and creases suggestive of crumpling (figure 2*a*,*f*). Most specimens bear minute cuticular specializations on their surface. Small spinules are observable on at least 7 coniform sclerites (figure 2*c–f,i,o,r–s*). In addition, 7 coniform sclerites show sparse perforations on their outer surfaces (figure 2*a*,*c–i,r*). The perforations are ellipsoidal (approximately 2–7 μm wide and approximately 6–10 μm long) and appear randomly distributed, except for one specimen where they align in two parallel rows along the height of the coniform sclerite (figure 2*h*). Four specimens also preserve a mesh of delicate, hexagonally patterned basal cuticle, which fades gradually into the smooth and more robust wall of the apical region (figure 2*a–b,e,h,i*) and is most conspicuous on the largest coniform sclerite found (figure 2*a*,*b*).

#### (iii) Arborescent structures

Arborescent cuticular structures are found physically associated with the bases of the hook-like (figure 1*a–b,d–h,l,n–r,t–u*) and coniform sclerites (figure 2*h*, *j*–m,o–p) of *Scalidodendron*, but also occur tightly packed together (with or without associated sclerites) in dense aggregates (figure 3*d,m–n,o–p,u–y*; *n* = 15). These aggregates can form extensive mat-like structures up to approximately 300 wide (figure 2*j*, 3*f,l,v-w*). By comparison, each individual arborescent structure reaches a maximum length of approximately 50 μm (figure 3*p*,*r*,*z*). The arborescent structures always comprise a ‘crown’ of branching projections, and a strap-like cuticular ‘shaft’ <5 μm thick. The shafts have a blunt, occasionally truncate termination (figure 3*o*), and splinter into the branching projections at the opposite extremity (figure 3*p*).

Arborescent structures are found attached to 12 out of 27 sclerites belonging to *Scalidodendron*, and the association was found to be statistically significant at *p* < 0.001 using a Fischer exact test (electronic supplementary material, data S1). By contrast, they were never found associated with any of the other 926 SCFs recovered from the Hess River assemblage (electronic supplementary material, data S1). These data suggest that the recurrent association of *Scalidodendron*’s sclerites and arborescent structures is unlikely to be due to chance, pointing to an original connection between these element types.

Besides these basic similarities in their architecture and positioning, the arborescent structures show some morphological variation depending on their placement relative to the co-occurring sclerites. Those found in isolated ‘thickets’ or associated with coniform sclerites (‘Type 1’ structures; figures 2*l-m,p* 3*d-e,j,l,o-p,w-y*) tend to display the greatest complexity. Type 1 specimens have a palmate shape, with up to four main branches radiating outwards and distally from the apex of the shaft (figure 3*i*,*k*,*o*–*p*,*r*,*t,z*). In turn, each branch splits recursively into up to five orders of finer bifurcations (figure 3*s*). These additional splits are usually dichotomous; some threefold splits are observed in distalmost regions (figure 3*t*). The distalmost splits of Type 1 structures produce exceptionally delicate and elongate cuticular tendrils up to approximately 40 μm long (figure 3*i*,*p*,*z*). In addition to these distal extensions, Type 1 structures may bear three laterally projecting cuticular rods of constant size. As shown by their variable orientation, the rods were loosely anchored at one end in a ‘cantilever’ fashion, and free to pivot around this attachment point approximately halfway along the length of the shaft (figure 3*k*,*n*,*p*).

The arborescent structures clustering around hook-like sclerites (‘Type 2’ structures; figures 1*a*,*g*,*o*,*q* and 3*a*,*b*) tend to exhibit a simpler morphology than Type 1 counterparts, lacking finely splintering crowns of bifurcating tendrils. Their palmate branches simply taper to a fine point distally, and show distinctive bilaterally fringed or ‘serrate’ margins (figures 1*b*,*h*,*r* and 3*b*). Possible laterally projecting rods were only observed in one cluster of Type 2 structures (figure 1*h*), suggesting that they were more infrequent or loosely anchored in this morphotype.

The morphology and placement of the arborescent structures suggest that they record original cuticular features associated with the hook-like and coniform sclerites, rather than exogenous biological material or debris. Some microbial microfossils from Neoproterozoic and Cambrian strata are characterized by branching patterns ([25], fig. 3, 5B; [26]), exemplified by *Pseudodendron* ([27], figs. 21, 28) and *Baltinema* ([19], fig. 11). However, none shows the rigid shaft, morphologically constrained palmate branching, serrate margins, tri-axial lateral ‘rods’ of constant length, or degree of recursive apical splitting expressed by the arborescent structures. The same is true of the organic fossils of cyanobacterial filaments [25,28]. Moreover, the arborescent structures are not borne on the spinules, uncinate terminations, or comb-like projections of *Scalidodendron*’s sclerites, as would be expected if they were exogenous items trapped and retained by these protruding cuticular outgrowths. Instead, they cluster around the base of the sclerites (figure 1*a*,*d*,*f*–*g*,*l*–*p,q,t*, figure 2*h*,*j*,*o,q*), suggesting an original placement on the animal’s body wall.

An original anatomical connection between the arborescent structures and associated sclerites is also supported by their consistent co-orientation and apparent morphological continuity. The arborescent structures become progressively smaller and less elaborate (in the form of simple bifid spines; figures 2*p* and 1*u*) closer to (and above) the base of associated hooked and coniform sclerites, and appear to grade smoothly into the unbranched spinulose and comb-like projections adorning their surface more distally (figures 2*p* and 1*t*). When adpressed onto the base of hook-like or coniform structures, the arborescent projections also tend to orient so that their branching terminations point towards the apex of these sclerites (figures 1*g*,*e–f,u* and 2*p*). Such an orientation is most evident in semi-articulated specimens where the arborescent structures display the most orderly and consistent arrangements (figure 1*a*,*p*). This suggests that the blunt ends of the arborescent structures originally represented their bases, whereas their branching projections extended apically and away from the body surface.

## 3. Discussion

The disarticulated nature of SCFs, freed from the host matrix and superimposed body parts and preserving organic structures down to submicron scales, permits the reconstruction of fossilized cuticular morphologies in unparalleled detail [18,20,29]. At the same time, it means that the biological properties of these fossils, including their homology and function, must be ‘reassembled’ from first principles or by comparison with similar structures in articulated specimens, where their positioning and anatomical connections are better-resolved. For structures lacking obvious counterparts in living or macrofossil taxa, as in the case of *Scalidodendron* SCFs, both approaches can be combined to reduce uncertainty on complementary aspects of an organism’s phylogenetic affiliation, autecology and broader-scale palaeobiological significance.

### (a) Phylogenetic affinities

**Figure 4.**
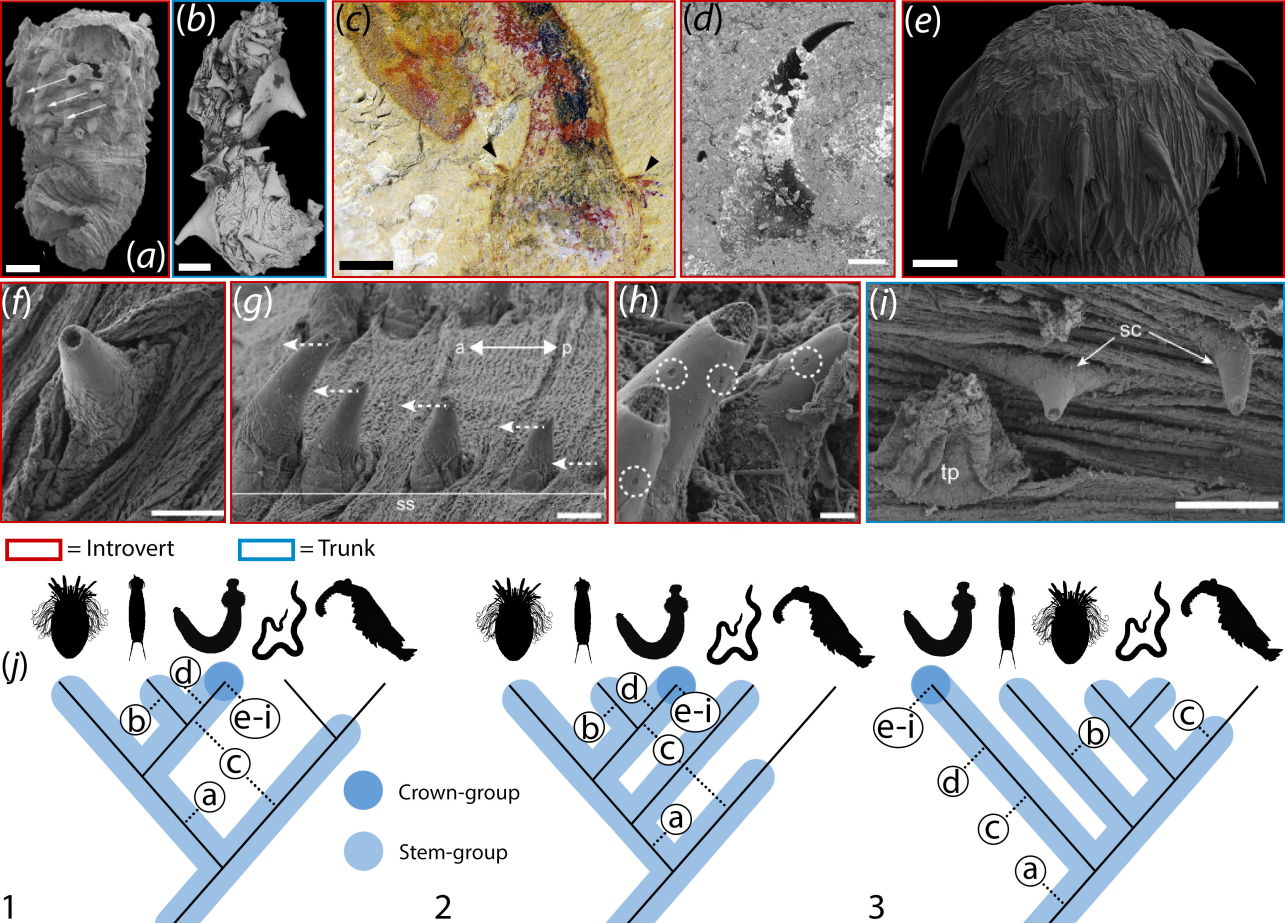
Putative phylogenetic placement and comparative morphology of *Scalidodendron*. (*a–h*) Hook-like and coniform sclerites in representative scalidophorans. (*a*) *Eopriapulites sphinx*, a potential stem-group scalidophoran; lateral view showing oblique alignment of introvert scalids (arrows) (reproduced with permission from [3]). (*b*) Fragmented trunk of *Eokinorhynchus rarus*, a potential stem-group kinorhynch, showing recurved trunk spines (from [4], reproduced under an Open Access Creative Commons CC BY license). (*c*) Introvert hooks (arrowed) from the palaeoscolecid *Tabelliscolex*, considered a stem-group priapulid or panarthropod (from [30], reproduced under an Open Access Creative Commons CC BY license Deed - Attribution 4.0 International - Creative Commons). (*d*) Introvert hook from the stem-group priapulid *Ottoia* (from [16], reproduced under a Creative Commons CC BY license). (*e*) Priapulid larva (*Priapulus caudatus*) showing recurved, radially arranged introvert hooks. Image courtesy of Graham Budd. (*f*) Coniform scalid from the circumoral field of the introvert of *P. caudatus*. (*g–h*) Lateral (*g*) and frontal (*h*) views of introvert scalids (ss) in *P. caudatus*; double arrow shows orientation of anterior (*a*) and posterior (*p*) of specimen. Dotted arrows show orientation of subapical depressions (circled). (*i*) Coniform scalid (sc) next to a trunk papilla (tp) on the anterior trunk of *P. caudatus*. Images f-i from [31], reproduced under an Open Access Creative Commons CC BY license Deed - Attribution-NonCommercial 4.0 International - Creative Commons. The anatomical position of structures in a–i is denoted by colour-coded legend (bottom). (*j*) Simplified trees of Ecdysozoa based on [6] (1) and [7] (2-3), showing the placement of (left to right in 1) loriciferans, kinorhynchs, priapulids, nematoids and panarthropods. Plausible placements of *Scalidodendron* under different proposed scenarios of ecdysozoan interrelationships are schematically represented by the colour-coded legend (bottom). Proposed phylogenetic placements of the taxa shown in *a–i* are denoted by their respective letters inside white circles. Taxon images sourced from phylopic.org. under CC0 1.0 Universal Public Domain Dedication licences (https://creativecommons.org/publicdomain/zero/1.0/). Scale: a-b, d, i, 100 μm; c, 1 mm; f-g, 50 μm; e, h, 10 μm.

The hook-like and coniform sclerites of *Scalidodendron* can be attributed to a scalidophoran-type animal (figure 4). Although Cambrian molluscs produced hollow organic sclerites [32], a lophotrochozoan affinity for *Scalidodendron* is challenged by the absence of the microvillar construction of fine longitudinal fibres diagnostic of this superphylum [20,33–35], which contrasts with the smooth, continuous texture of the walls of coniform and hook-like sclerites (figures 1, 2; cf. electronic supplementary material, fig. S5b–c). By contrast, a fibrous microstructure is readily identifiable in Cambrian molluscan, brachiopod and annelid SCFs [19,34] and macrofossils [32]. Similarly, a chaetognath producer would be inconsistent with the thin-walled construction, subcircular cross-sections and large internal cavities of *Scalidodendron* SCFs. These features contrast with the laterally compressed profile, thinner pulp cavity, narrow keel, internal fibrous microstructure [36] and dense chitinous walls of chaetognath grasping spines (electronic supplementary material, figure S5a), which also lack the pervasive spinulose ornamentation of *Scalidodendron* [37].

The morphology of *Scalidodendron* SCFs is also at odds with known non-scalidophoran ecdysozoan producers. Unlike the solid cuticular spines of nematoids [4,36], the sclerites of *Scalidodendron* are hollow. They also differ markedly from the straight-sided spines of hallucigeniids which, although covered by scale-like ornamentation, have a distinctive layered ‘cone-in-cone’ construction [38]. The circumoral cuticular elements and aciculate pharyngeal teeth of panarthropods (including Cambrian lobopodians) also differ from *Scalidodendron*’s hollow and recurved sclerites in their lamellate or stylet-like morphology [39]. By contrast, hollow organic-walled sclerites comparable to those of *Scalidodendron* are characteristic of scalidophorans [4,40]. Such structures have been widely documented in Cambrian priapulids [16], putative stem-group kinorhynchs [4] and stem-group scalidophorans [3,41,42].

Besides matching scalidophoran elements in their overall architecture, the morphologies of particular types of *Scalidodendron* SCFs map closely onto forms observed in extant and fossil members of the group. In particular, *Scalidodendron*’s hollow, smooth-walled coniform sclerites with an apical and a basal opening find close counterparts in the cuticular cones of living priapulids ([8], fig. 10; [31], figs. 4–5, 12E). Ovoid fenestrations similar to those of *Scalidodendron* in size, shape and positioning occur on similar scalidophoran ‘cones’ from the early Cambrian File Haidar formation of Baltica ([19], fig. 7I–M) and the latest middle Cambrian Pika Formation of Alberta [43]. In addition, rounded subapical ‘depressions’ comparable in shape and positioning to the fenestrations of *Scalidodendron* have been documented in the extant priapulid *Priapulus caudatus* [31] (figure 4*h*). The falciform hook-like sclerites of *Scalidodendron* (figure 1) are also similar in shape, size and aspect ratio to the hooks found on the introvert [41] or trunk [4] of putative Cambrian stem-group scalidophorans and kinorhynchs, and on the introvert of fossil and living priapulids [2,44]. Similar falcate, hook-like sclerites were borne on the introvert [30,45] and occasionally the trunk [46] of Cambrian palaeoscolecids [5,30,47,48].

A scalidophoran attribution is corroborated by the patterned cuticles occasionally extending around the base of *Scalidodendron*’s sclerites. Cuticles patterned into microreticulate meshes of hexagons (figure 2*a,b*) are found in extant priapulids, such as *Priapulus* and *Halicryptus* ([49], fig. 2; [50], fig. 2). As in *Scalidodendron*, in modern priapulids hexagonally patterned cuticles may adorn the base of coniform sclerites (figure 2*a,b*; cf [8], fig. 10). Hexagonally patterned cuticle is also found attached to the sclerites and pharyngeal teeth of Cambrian priapulids [16,19,43], and on the trunks of palaeoscolecid worms ([51], fig. 4R) and putative stem-scalidophorans [50,52].

Given the broad phylogenetic distribution of hook-like and coniform sclerites among scalidophorans (figure 4), and the status of palaeoscolecids as possible stem-group relatives of priapulids, scalidophorans, nematoids, panarthropods and/or Ecdysozoa as a whole [5,30,47,48], the affinities of *Scalidodendron* (figure 4) ultimately lie in a broad plexus of ‘scalidophoran-grade’ animals encompassing the priapulid, kinorhynch and loriciferan lineages, and potentially the stem-group relatives of panarthropods, nematoids and all ecdysozoans [6,7].

### (b) Arborescent structures: positioning and morphological comparisons

While the attribution of *Scalidodendron*’s coniform and hook-like sclerites to a scalidophoran-grade animal appears secure, the associated arborescent structures lack similarly unambiguous counterparts in known taxa. Nonetheless, their positioning can be inferred based on their association with less exotic body structures. In known scalidophorans, coniform and hook-like sclerites—comparable to those associated with the arborescent structures (figure 1)—occur on the introvert or on the trunk [2,3,8,44,53–55]. Introvert scalids in priapulids, kinorhynchs and loriciferans are thought to be involved in locomotion [2,55,56]; hook-like trunk sclerites may contribute to anchorage, and potentially physical defence [12,57–60]. By contrast, coniform structures borne on priapulid introverts ([8], fig. 10; [31], figs. 4–5) or trunks ([31], fig. 12E) house adhesive or sensory tubuli [31]. These sclerites lie posterior to the feeding pharynx, suggesting the same positioning for the closely associated arborescent structures of *Scalidodendron*.

A post-pharyngeal position for the arborescent structures and their strap-like to filiform shapes invite comparison with the hairlike projections found on the trunk of other scalidophorans. The trunk of the Burgess Shale priapulid *Ancalagon* ([13], plate 26) bears densely spaced simple setae, forming a uniform pilose covering that superficially recalls the arrangement of the Hess River arborescent structures. Unlike in *Scalidodendron*, however, the hair-like extensions of *Ancalagon* do not show branching or lateral projections, and thus offer no direct morphological counterparts to the Hess River SCFs.

Among living taxa, cuticular ‘hairs’ cover the trunk segments of some kinorhynchs [61,62], forming a dense cover on the external body surface [63,64]. Once again, however, these are unbranched, and insert in specialized ‘perforation sites’ [65]) not observed in *Scalidodendron*. Morphologically more complex cuticular projections occur among loriciferans. Notably, larval pliciloricids form up to five pairs of radially arranged setae on the anterior region of the lorica, each exhibiting up to two orders of asymmetric bifurcations. The comparison with *Scalidodendron* remains less than compelling, however: the distal ‘prongs’ of plicilorid setae are well-spaced and recurved, and without the finely splintered apices of the arborescent structures ([66,67] fig. 47).

Taken together, the marked differences between *Scalidodendron*’s arborescent structures and the hairs of priapulids, kinorhynchs and loriciferans show that these branching specializations lack direct morphological counterparts among any known scalidophorans.

### (c) Functional morphology of the arborescent structures

The close association of the arborescent structures with post-pharyngeal sensory or locomotory sclerites suggests that, like the cuticular ‘hairs’ of other scalidophorans, they did not contribute directly to feeding. What then might have been their function and, by extension, their autecological significance?

The setae of *Ancalagon* have been speculated to serve a sensory function; however, their lack of branching or lateral projections [12] hampers a direct functional comparison with *Scalidodendron*. Moreover, sensory structures in living scalidophorans (including mechano- and chemoreceptive scalids) contain bundles of innervated sensory cells communicating with the exterior via a distal pore [53,55,68,69]. To house these structures, specialized sensory projections in extant taxa have a tubular construction, exemplified by kinorhynch [54,55,70] and loriciferan [66] spinoscalids, priapulid peripharyngeal sensory scalids [53,68] and priapulid cuticular ‘cones’ ([8], fig. 10; [31], fig. 5). No distal pore or other openings were observed on the arborescent structures, and the presence of internal bundles of sensory cells would be difficult to reconcile with their strap-like morphology and distal tendrils splitting into fine, separate points [44,53–55,66].

The submicron-scale mesh of the arborescent structures might suggest a role in trapping or filtering microscopic prey. However, feeding structures in other scalidophorans—including the filamentous bristles of microphagous taxa [71], fig. 7D—are borne on the pharynx or its surrounding ring of scalids [2,17,55,72]. Notably, none of the Hess River arborescent structures were recovered in physical association with pharyngeal teeth (electronic supplementary material, figure S2), but instead exclusively with more posterior sclerites probably adapted for sensory, locomotory and/or defensive purposes.

An alternative respiratory function would be consistent with the finely branching morphology of the arborescent structures, which would have afforded high surface area-volume ratios for gas exchange. The arborescent structures share with all other ecdysozoan SCFs [20] a recalcitrant cuticle consisting of robust extracellular matrix. Cuticular respiratory surfaces occur in annelid branchiae [73], and extant scalidophorans are hypothesized to breathe through relatively thin cuticular integument—as found in priapulid caudal appendages [2,74]. Nonetheless, no living scalidophorans (or other known ecdysozoans) conduct substantial gas exchange through morphologically similar dendritic extensions distributed on locomotory regions of the body. A respiratory function would also leave unresolved the function of the serrations (figures 1*b* and 3*b*) and tri-axial laterally projecting rods of the arborescent structures (figure 3*k*,*n*,*p*), which lack surface area-enhancing dendritic splits.

A locomotory role also finds no obvious counterparts. The delicate arborescent structures differ markedly from the stiff, hook-like setae used as substrate ‘anchors’ by burrowing or bottom-dwelling invertebrates [75,76]. The bifurcating lorical setae of pliciloricids are much less dense or morphologically elaborate than the arborescent structures ([67], figs. 4, 39, 47, 49, 63), and operate in interstitial environments at scales incompatible with the plausible size range of *Scalidodendron*: the total body length of loriciferans [77] falls within the same order of magnitude as each of *Scalidodendron*’s individual sclerites (figures 1–2). Moreover, complex arborescent projections may hinder rather than facilitate locomotion: their branching morphology and extensive, densely packed tendrils would be expected to increase drag ([78]) and potentially trap solid obstacles, debris or sediment within their submicron-scale mesh.

The potential ‘particle trapping’ capabilities of *Scalidodendron*’s cuticular tendrils leave open the possibility of more exotic defensive or camouflaging functions. Sediment and debris-trapping projections have evolved many times convergently in ecdysozoans and other protostomes (electronic supplementary material). These structures capture and fasten substrate particles [79,80] or organic debris [81,82] from the surrounding environment, offering camouflage from visual predators and protection from physical insults. Debris and sediment-trapping setae vary greatly in size and morphology depending on the type, scale and density of the target ‘cloaking’ material [83,84]. Nonetheless, finely subdivided cuticular specializations are recurrent adaptations in the trunk of microdebris- and soil-capturing arachnids [85] and insects [79–81]. These debris- and sediment-trapping ‘splintered setae’ occur in terrestrial animals (electronic supplementary material), and as such offer no direct autecological counterparts to *Scalidodendron*. However, their structural parallels and comparable degree of elaboration (electronic supplementary material, figure S4) suggest functional similarities and a shared defensive role (electronic supplementary material).

Visual predation has been hypothesized as one of the key drivers of the trophic ‘arms races’ escalating during the Cambrian [86–89]. However, data on adaptations for visual camouflage among Cambrian fossils has so far been lacking. If they do record ‘cloaking’ devices, *Scalidodendron*’s arborescent structures would denote crypsis in an early-diverging ecdysozoan lineage (electronic supplementary material, figure S6), extending by around 90 million years [84] the record of this phylogenetically disparate driver of Phanerozoic predator-prey coevolution [90,91].

### (d) Implications for early scalidophoran disparity

Irrespective of their precise role—respiratory, sensory, feeding, defensive or a combination thereof— *Scalidodendron*’s exotic cuticular projections suggest a correspondingly unconventional function, mapping onto a morphology that falls beyond the range of variation of known scalidophorans. Compared to previously described scalidophoran sclerites or hairs, the arborescent structures display at least four additional nested orders of subdivisions (figure 3*s*). This reflects a degree of recursive substructure in cuticular architectures that among ecdysozoans is otherwise only known in arthropods [21,84].

Morphometric analyses of Cambrian scalidophoran disparity have largely sampled higher-order characters—such as the geometry or arrangement of scalids on the body—rather than individual sclerite morphologies, which are often too poorly preserved to permit their morphological reconstruction [92,93]. Alternatively, they have focused on isolated priapulid pharyngeal teeth, which express conserved morphological landmarks allowing secure quantitative comparisons [17]. Neither approach has detected patterns of higher disparity in Cambrian than modern forms; instead, they have suggested somewhat greater morphospace occupancy in the latter [17,92,93]. By contrast, the Hess River specimens suggest that the largely untapped SCF record [18,20] could yield a different picture at the level of finer-scale cuticular substructures, often obscured by alternative taphonomic pathways [3,17].

The recognition of exotic morphologies in Cambrian scalidophoran SCFs is consistent with previously documented ‘early burst’ disparity patterns in the history of metazoan lineages [22,94,95], whereby early appearing taxa approach or exceed the limits of morphospace occupancy of a given clade. Among Cambrian ecdysozoan worms, the most extreme of such patterns has been detected in onychophorans [22]. Lobopodian-grade stem-onychophorans show complex suspension feeding appendages, armours and spinulose ornaments unlike those of any living relative [22,23,38,96,97]. Unlike the cuticular adaptations of *Scalidodendron*, the exotic appendage and trunk structures of lobopodians do not consist of morphologically ‘exotic’ individual elements: instead, they combine simple unbranched setae, spines and tubercles into higher-order architectures lacking extant counterparts, as seen in the luolishaniid suspension-feeding ‘basket’ [22,23,96,97], the dorsal spine rows of hallucigeniids [38] or the spiny armour of *Diania* [96,98].

The exotic functional complexes of lobopodians manifest at the level of macroscopic anatomical units spanning individual limbs to entire body tagmata, far removed from *Scalidodendron*’s micrometre-sized specializations. This difference in scale showcases how different Cambrian ecdysozoan groups acted on nested yet distinct levels of organization to produce their unique morphological specializations. Notably, the combinatorial potential of modular limbs and metameres, leveraged by early members of the panarthropod phyla to assemble their functional complexes, would not have been available to Cambrian scalidophorans: members of the group show a relatively conservative tripartite bodyplan, lacking the repeated, differentially modifiable body units of onychophorans, tardigrades and arthropods [2,5,6]. In the escalatory context of Cambrian ecologies, scalidophorans could have outsourced morphological specialization to smaller-scale but similarly repeated and locally differentiable structures, including discrete hairs, teeth and spines. In this light, ‘cryptic’ high disparity among early scalidophorans would be consistent with the rapid occupation of new ecospace, and the attendant high rates of genotypic and phenotypic innovation [99], defining the Cambrian Explosion.

## 4. Material and methods

SCFs were extracted from a single field sample of shale from the Hess River Formation, at an unknown stratigraphic height. The closest published section to the study locality is §10 of [100]. The 100−200 grams sample was processed for SCFs following the procedure in [18]. A total of 968 SCFs representing multiple metazoan phyla and microbial problematica (electronic supplementary material, data S1) were recovered. Of these, 42 specimens were attributed to *Scalidodendron* (including the holotype and 41 paratypes) and 22 were referred to the same taxon (see §2). All specimens are reposited at the Geological Survey of Canada (GSC). Specimens were imaged using a Kontron Elektronik ProgRes 3012 camera fitted to a Zeiss Axioplan 2 stereomicroscope. Images were assembled in Adobe Photoshop 2024 using focus stacking. To test for a significant association between arborescent structures and *Scalidodendron*’s sclerites under the small (<1000) available sample size [101], Fischer’s two-tailed exact test was performed in Microsoft Excel using the HYPGEOM.DIST formula (electronic supplementary material, data S1).

## Data Availability

Our fossil specimens catalogue can be accessed in our open access electronic supplementary material [102].
